# A New Species of *Dialictus* from Sombrero Island, Anguilla (Hymenoptera, Halictidae)

**DOI:** 10.3897/zookeys.86.909

**Published:** 2011-03-19

**Authors:** Michael S. Engel

**Affiliations:** Division of Entomology, Natural History Museum, and Department of Ecology & Evolutionary Biology, 1501 Crestline Drive – Suite 140, University of Kansas, Lawrence, Kansas 66049–2811, USA

**Keywords:** Apoidea, Anthophila, Halictidae, Halictinae, Halictini, taxonomy, Anguilla, West Indies

## Abstract

A new species of *Lasioglossum* Curtis subgenus *Dialictus* Robertson (Halictinae, Halictini) is described and figured from a series of female and males collected on Sombrero Island, Anguilla; the northernmost island of the Lesser Antilles. *Lasioglossum (Dialictus) sombrerense* **sp. n.** is distinguished from its congeners and the name made available for a forthcoming work on the arthropod diversity of Sombrero Island.

## Introduction

Since the overview by Eickwort (1988), work has steadily increased on the West Indian halictine fauna (e.g., Snelling 2005; Genaro 2006, 2007, 2008; Genaro and Franz 2008; Smith-Pardo 2009) and it is greatly hoped that a new synthesis of this entire region is undertaken. The purpose of the present contribution is to provide a name for a new species of halictine bee on Sombrero Island, Anguilla. The name is established here so that it may be used in a forthcoming study of Sombrero Island arthropod diversity by Mike Ivie and Justin Runyon (Ivie and Runyon, in prep.).

Institutional acronyms used in the sections on the type material are: SEMC, Snow Entomological Collection, Division of Entomology, University of Kansas Natural History Museum, Lawrence; NHML, Department of Entomology, The Natural History Museum, London; FSCA, Museum of Entomology, Florida State Collection of Arthropods, Gainesville; and MTEC, Montana Entomology Collection, Montana State University, Bozeman. Morphological terminology generally follows that of (Engel (2001, 2009) and Michener (2007), while the format for the description loosely follows that of Engel (2000) and Engel et al. (2007). Measurements were taken using an ocular micrometer on an Olympus SZX-12 stereomicroscope while photomicrographs were prepared with a Nikon D1x digital camera attached to an Infinity K-2 long-distance microscopic lens. Measurements are of the holotype, with ranges for paratypes provided in parentheses.

## Systematics

**Genus *Lasioglossum* Curtis**

**Subgenus *Dialictus* Robertson**

### Lasioglossum (Dialictus) sombrerense

Engel sp. n.

urn:lsid:zoobank.org:act:380A073D-2F45-4933-9B64-697690DF6D03

Figs 1 [Fig F2] 9 

#### Holotype.

♀, Sombrero, West Indies: 18°35'11.4"N, 63°25'37.8"W, 12 November 1999, M.A. Ivie & J.B. Runyon (SEMC).

#### Paratypes.

5♀♀ Sombrero, West Indies: 18°35'11.4"N, 63°25'37.8"W, 12 November 1999, M.A. Ivie & J.B. Runyon (SEMC, MTEC, NHML); 5♀♀1♂, Sombrero, West Indies: 18°35'10.26"N, 63°25'37.86"W, 12–13 November 1999, M.A. Ivie & J.B. Runyon (SEMC, MTEC, FSCA).

#### Diagnosis.

**Figures 1–3.**
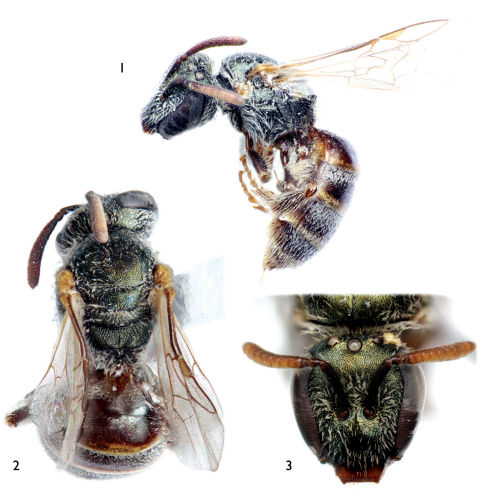
Female of *Lasioglossum (Dialictus) sombrerense* Engel, sp. n. **1** Lateral habitus **2** Dorsal habitus **3** Facial aspect.

**Figures 4–6. F2:**
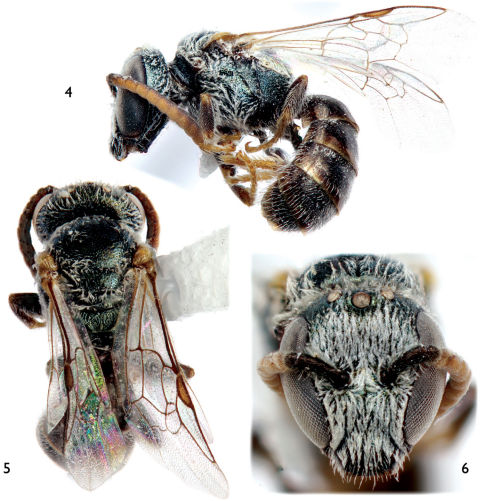
Male of *Lasioglossum (Dialictus) sombrerense* Engel, sp. n. **4** Lateral habitus **5** Dorsal habitus. **6** Facial aspect.

**Figures 7–9.**
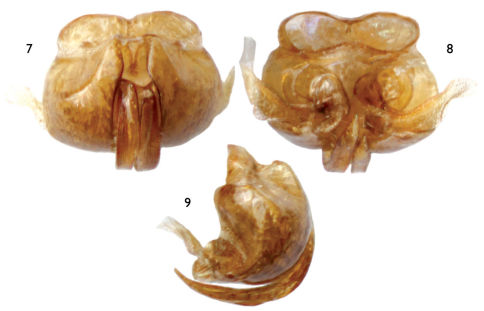
Male genitalia of *Lasioglossum (Dialictus) sombrerense* Engel, sp. n. **7** Dorsal aspect **8** Ventral aspect **9** Lateral aspect.

Small bees, around 5 mm or less in total length; integument of head and mesosoma dark metallic green with bluish and coppery tints in places (Figs 1–5); apical half of clypeus of male and female dark brown to brown (Figs 3, 6); flagellum two-toned, brown to dark brown above, honey brown below (Figs 1, 3, 4, 6); female metasoma with abundant appressed tomentum on metasomal terga III–V and basally and laterally on tergum II (Fig. 1); male face densely covered with plumose appressed to subappressed setae (Fig. 6); and male genitalia as in figures 7–9.

#### Description.

##### Female:

Total body length 4.93 mm (4.03–5.00 mm); forewing length 2.83 mm (2.53–2.86 mm). Head longer than wide, length 1.32 mm (1.23–1.34 mm), width 1.22 mm (1.13–1.22 mm); upper interorbital distance 0.77 mm (0.73–0.77 mm); lower interorbital distance 0.66 mm (0.60–0.66 mm). Mandible with weak subapical tooth; labrum with weak apical callosity; majority of clypeus below lower tangent of compound eyes; malar space linear. Intertegular distance 0.89 mm (0.81–0.91 mm); mesoscutellum slightly longer than metanotum, about as long as basal area of propodeum. Forewing with distal venation weakened (1rs-m, 2rs-m, and 2m-cu); basal vein 2–2.5 times distad cu-a; combined lengths of second and third submarginal cells slightly less than length of first submarginal cell; second submarginal cell slightly narrowed anteriorly, anterior border along Rs about as long as that of third submarginal cell along Rs; hind wing with distal hamuli arranged 2-1-2. Inner metatibial spur with three distinct branches distributed along rachis of spur and decreasing in size and angle relative to rachis from proximal to apical, sometimes with a minute fourth branch apically and poorly differentiated from rachis.

Mandible outer surface smooth with scattered minute punctures; labrum smooth with scattered minute punctures, punctures more sparse leading up to apical callosity; clypeus with apical half smooth and with sparse shallow coarse punctures, basally faintly imbricate with small punctures separated by 1–2 times a puncture width; supraclypeal area imbricate with small punctures separated by a puncture width except along border with clypeus integument between punctures smooth to faintly imbricate; face below level of antennal toruli imbricate with small punctures, punctures separated by less than a puncture width to nearly contiguous except bordering compound eye and clypeus integument smooth to faintly imbricate and punctures separated by a puncture width or less; face above level of antennal toruli with small punctures separated by less than a puncture width, integument between punctures, where evident, faintly imbricate; punctures separated by a puncture width or less in parocular area and between lateral ocelli, integument between punctures smooth; vertex with scattered small punctures; gena with small punctures separated by a puncture width or less, integument smooth to faintly imbricate; postgena imbricate. Pronotum imbricate; mesoscutum imbricate with small punctures separated by a puncture width or less, such punctures more faint anteromedially and becoming separated by 1–2 times a puncture width; punctures outside of parapsidal lines separated by a puncture width or less; punctures along posterior border separated by less than a puncture width; mesoscutellum imbricate with small punctures separated by 1–2 times a puncture width on disc, punctures more closely spaced along borders; metanotum with minute punctures separated by less than a puncture width, integument between punctures apparently smooth; pleura with small punctures separated by less than a puncture width in hypoepimeral area, integument between punctures smooth to faintly imbricate; remainder of pleuron with slightly larger punctures separated by less than a puncture width although becoming more widely spaced ventrally and ventroposteriorly, integument between punctures faintly imbricate; metepisternum faintly imbricate to imbricate with punctures separated by less than a puncture width; basal area of propodeum strongly imbricate to nearly granulose, with irregular striae radiating from basal margin, such striae stronger laterally, medially becoming more rugulose apically; lateral surface of propodeum smooth to faintly imbricate with small punctures separated by less than a puncture width; posterior surface of propodeum imbricate. Metasomal terga faintly imbricate with scattered minute shallow punctures, punctures separated by 2–3 times a puncture width, becoming sparser and fainter in apical margins; sterna faintly imbricate.

Integument of head and mesosoma dark metallic green, with some faint bluish and or coppery tints except labiomaxillary complex and labrum dark brown; apical half of clypeus brown to dark brown; mandible dark brown at base, blending to honey brown medially, then to reddish brown at apex; scape, pedicel, and first flagellomere dark brown, remainder of flagellum brown to dark brown above and honey brown ventrally. Pronotal lobe honey brown; tegula honey brown and semi-translucent; legs dark brown except lighter on tarsi and at femorotibial joints. Wing membranes hyaline, veins generally honey brown except C, Sc+R, and R distad pterostigma brown. Metasoma generally dark brown except apical margins of terga semi-translucent and lighter brown, pseudopygidial area lighter brown; sterna brown to dark brown with narrow lighter apical margins.

Pubescence generally white (Figs 1–6). Head and mesosoma with scattered short to long, branched, suberect to erect setae, not obscuring integument; such setae more numerous on face around and below level of antennal toruli, and on vertex, gena, pronotal dorsal ridge, around pronotal lobe, on metanotum, pleura, and lateral and posterior surfaces of propodeum. Metasomal terga III–V with abundant plumose, appressed tomentum (Fig. 1), similar tomentum on basally and laterally on second metasomal tergum, first metasomal tergum without such tomentum but with sparse, suberect to erect setae, such setae minute over disc, becoming longer laterally, such setae appressed and posterolaterally-directed basally on anterior-facing surface; such suberect to erect setae scattered on remaining metasomal terga but most prominent and numerous laterally and on more apical terga; sterna with areas of longer erect setae apically on discs and margins.

##### Male:

As described for the female except in usual gender differences and as follows: Total body length 5.17 mm; forewing length 2.63 mm. Head longer than wide, length 1.21 mm, width 1.09 mm; upper interorbital distance 0.68 mm; lower interorbital distance 0.46 mm. Mandible simple; labrum transverse, without callosity; apical half of clypeus below lower tangent of compound eyes. Intertegular distance 0.73 mm. Forewing second submarginal cell with anterior border along Rs much shorter than that of third submarginal cell along Rs. Inner metatibial spur simple. Male genitalia as in figures 7–9.

Face below level of antennal toruli imbricate with small punctures, punctures separated by less than a puncture width to nearly contiguous; face above level of antennal toruli with small punctures separated by less than a puncture width, integument between punctures, where evident, faintly imbricate or smooth; punctures separated by less than a puncture width in parocular area and between lateral ocelli, integument between punctures smooth. Punctation of mesosoma coarser than in female; mesoscutum imbricate with small punctures separated by a puncture width, such punctures more faint anteromedially and becoming more widely spaced; punctures outside of parapsidal lines and along posterior border separated by less than a puncture width.

Mandible largely honey brown with reddish brown apex; scape and pedicel dark brown, flagellum brown to dark brown above and honey brown ventrally.

Face densely covered in appressed to subappressed, plumose setae, largely obscuring integument (Fig. 6); metasomal terga without abundant plumose, appressed tomentum (Fig. 4) except for thin patches basolaterally on II and III.

#### Etymology.

The specific epithet is based on the name of the island to which the species was captured, Sombrero Island (a.k.a. Hat Island), Anguilla, West Indies, northernmost island of the Lesser Antilles.

#### Comments.

The new species has some similarities to the widespread *Lasioglossum (Dialictus) parvum* (Cresson). Both are of relatively similar proportions and coloration but the antenna in *Lasioglossum parvum* is not so strikingly two-toned, the integument tends to have a more bluish cast, the apex of the clypeus is more dark brown, the abdomen lacks the dense covering of tomentum observed in *Lasioglossum sombrerense* (e.g., Fig. 1), the mesoscutal punctures are more widely spaced (separated by a puncture width or more on disc), and the propodeum is much more strongly rugosostriate throughout and reaching to the margins, particularly laterally where such striae extend onto the upper portions of the lateral surfaces. In other small West Indian species such as *Lasioglossum (Dialictus) busckiellum* (Cockerell), the abdomen in darker with reddish brown apical margins to the terga, the tegulae are reddish brown, the apical abdominal terga are not obscured by tomentum, the head is broader, and the propodeum has a finer file-like striation (Cockerell 1915), and in *Lasioglossum (Dialictus) gundlachii* (Baker) the punctation is finer and more widely spaced (Baker 1906).

## Supplementary Material

XML Treatment for Lasioglossum (Dialictus) sombrerense
